# Consumer Assessment of Pork Loin Quality: How Important Are Sensory Attributes, Pig Breed, and Familiarity?

**DOI:** 10.3390/foods14152587

**Published:** 2025-07-23

**Authors:** Ángel Millán, Marta Retamosa

**Affiliations:** 1 Marketing Department, Facultad de Derecho y Ciencias Sociales, Universidad de Castilla-La Mancha, Ronda de Toledo, 13003 Ciudad Real, Spain; angel.millan@uclm.es; 2 Marketing Department, Facultad de Ciencias Jurídicas y Sociales, Universidad de Castilla-La Mancha, Cobertizo San Pedro Mártir s/n, 45071 Toledo, Spain

**Keywords:** pork loin, quality, sensory attributes, breed, familiarity, latent class cluster analysis

## Abstract

The literature on pork quality perception is fragmented, particularly regarding the role of sensory and intrinsic attributes and consumer familiarity. This study addresses this gap by examining the importance of sensory attributes (juiciness, flavor, aroma, and tenderness) and an intrinsic attribute (pig breed—related to differences in color and fat content) in the overall quality assessment of pork loin. Additionally, it investigates how consumer familiarity with the pork loin category influences perceived quality. An experimental study was conducted with 130 Spanish consumers. The proposed hypotheses were tested using analysis of covariance (ANCOVA) models and a latent class cluster analysis to explore both the impact of specific attributes on perceived quality and the segmentation of consumers based on familiarity. The findings indicate that flavor, tenderness, and juiciness are the key sensory attributes influencing the overall quality perception of pork loin. In contrast, Duroc pork loin is perceived as being of lower quality. The cluster analysis identified three distinct consumer segments based on their level of familiarity with the product. This study contributes new empirical evidence to the understanding of the perceived quality of pork loin, highlighting the significant role of specific sensory attributes and consumer familiarity. These insights can inform product development and marketing strategies tailored to different consumer profiles.

## 1. Introduction

The meat processing sector is an extraordinarily responsive economic sector [1], and as a result, companies in the food sector face specific issues in their supply chains, such as perishability, shelf life, traceability, food quality and safety, seasonal behaviors, transport and storage conditions, as well as sustainability requirements. Increasing consumer attention to various meat alternatives has led the meat industry to make a number of structural and technological changes in order to remain a viable and competitive industry in the long term. In addition to attractive prices, consumers generally expect high-quality meat products, high manufacturing standards in terms of safety and hygiene, consideration of local and regional culinary traditions, and other factors [2]. Pork consumption trends vary significantly from country to country. Between 2010 and 2020, a decline in pork consumption was observed in various nations, including many European countries, Canada, and China. In contrast, consumption increased in regions such as Russia, the United States, Australia, New Zealand, most Central and South American countries, and some African nations [3]. According to OECD/FAO [4] projections, the trend from 2021 to 2030 indicates a continued decline in pork consumption in Europe and most developed countries, with growth expected in Latin America and several Asian countries. The sensory properties of a food product collectively contribute to the formation of positive expectations before and during consumption, but some attributes play a more prominent role than others in the overall evaluation [5]. At the individual level, taste or sensory appeal, likes and dislikes, and consumption patterns are significant in the selection and evaluation of foods [6]. Nevertheless, if consumer acceptance is a high priority for meat product development, the emphasis should be less on the technological aspects of meat and more on how consumers react to variations in sensory qualities, such as juiciness, flavor, aroma, or tenderness. A useful method for capturing hedonic responses related to these properties is the use of “check all that apply” (CATA) terms [5]. In this format, participants are presented with a list of descriptors (generated by a trained sensory panel or derived from the existing literature) and must select all those that they consider applicable to each sample evaluated. Thus, this method allows for estimating the perceived intensity of specific attributes by analyzing the frequency with which they are selected by consumers.

Most of the research conducted to date is isolated and fragmented. Specifically, previous research focuses on either the consumer studies (attitudes or purchases) or sensory science (consumer experience) domains. Combining methodologies from these two disciplines allows researchers to better understand consumer responses and determine whether current product features must be modified to increase the appeal of the product to the target consumer. This study attempted to overcome this limitation by employing a research methodology that combines both approaches (consumer attitude and purchase/sensory analysis).

Banović et al. [7] and Borgogno et al. [8] suggest that different levels of familiarity with meat products could be responsible for the differences in quality assessment. To the best of our knowledge, the existing studies do not comprehensively analyze this issue in the specific case of the pork loin product category. Our study aims to fill this research gap.

This study evaluates the importance of different sensory attributes (juiciness, flavor, aroma, and tenderness), along with that of an intrinsic attribute (pig breed, which determines differences in fat content and color), on the overall quality of pork loin, as perceived by consumers. Furthermore, it analyzes whether there are different consumer segments according to their familiarity with this product category, in addition to the influence of familiarity on perceived overall quality and its moderating effect on the relationship between breed and perceived quality.

### 1.1. Quality in the Meat Industry

The concept of quality in the meat industry is extensive and encompasses a wide range of product characteristics, including microbiological, technological, nutritional, and sensory attributes. Nevertheless, from the consumer’s perspective, meat quality is subjective and can differ among individuals, societies, and cultures. Therefore, as expected, the study of quality is complex, given the broadness of its concept [9].

According to previous findings, it is necessary to focus on the three main types of quality attributes discussed in the literature on consumer behavior (Table 1): search, experience, and credibility.

Not all the quality cues evaluated when purchasing and consuming are known. Moreover, these cues vary in their predictive value, which is the extent to which attributes contribute to consumer satisfaction. Search cues are used to predict experience and credence quality, and experience cues may be used to predict credence quality [12].

### 1.2. Quality Attributes of Pork Meat

At the point of purchase, consumers generally rely on extrinsic cues, such as color, visible fat, cut, packaging, and price, to determine which meat products to buy. Therefore, only a few extrinsic indicators are available because fresh meat is largely unbranded [13].

Eating quality is the most relevant factor in a consumer’s choice of meat [14]. Good eating quality includes the right combination of tender and juicy meat, along with an intense meat flavor [15]. Similarly, Garmyn [16] establishes that tenderness, juiciness, and flavor continue to be the three fundamental drivers of the palatability of cooked meat, all of which are directly associated with consumer satisfaction.

Moeller et al. [17] revealed that consumers’ overall acceptance of pork is mainly associated with color and tenderness, followed by flavor intensity and juiciness. Miller [18] shows that pH, water-holding capacity, tenderness, intramuscular fat (IMF), and final cooking temperature influence consumer preferences. The value of taste for consumers is evident; therefore, satisfying their taste expectations is one of the most important keys to success. Nevertheless, taste is a characteristic of the experience that varies not only among consumer segments, but also among product types [19].

#### 1.2.1. Color

Color is one of the most relevant attributes of fresh meat at the point of sale [20,21], because consumers use inappropriate color as an indicator of deterioration and unhealthiness [22]. Buyers accustomed to meat consumption often use color as an extrinsic attribute to anticipate the sensory quality [7,23].

For pork, darker and reddish colors are considered more attractive, although color preferences are highly conditioned by cultural differences [20,24]. Meanwhile, Troy and Kerry [23] conclude that consumers prefer pink pork. Grunert et al. [25] examined the different attributes of pork in an experimental study of German and Polish consumers and found that consumers in both countries preferred slightly pink- to red dark-colored meat.

#### 1.2.2. Fat Content

Another factor that affects consumers’ acceptance of pork is the amount of IMF it contains [26]. Previous studies have not fully clarified the relationship between the fat content of meat and its acceptability to consumers. Cannata et al. [27] reported a positive relationship between pork acceptability and IMF and/or marbling. In contrast, Moeller et al. [17] found that heavily marbled loins were less acceptable to consumers than low-marbled loins.

Nevertheless, a decrease in the level of fat in meat could negatively affect food satisfaction, as this parameter directly affects the palatability, flavor, and overall taste of meat [28]. Therefore, it is necessary to determine whether consumers notice changes in fat percentages, as low-fat meat may be considered a healthier product. If the sensory characteristics are negatively influenced, the product has no chance of success [29].

Regarding this topic, Font-I-Furnols [26] demonstrated that consumers’ visual appreciation of fresh pork loin slices with different levels of IMF/marbling was the most perceptible and important characteristic that affected the purchase decision. Based on this result, two consumer segments were identified as “marbled loin lovers” and “lean loin lovers”.

#### 1.2.3. Juiciness

Consumers prefer more tender and sometimes juicier pork meat [30]. Pork juice is one of the most important quality attributes for consumers and is determined by the quality of raw meat, cooking procedure, temperature, pH, and IMF content [31]. Rearing conditions can also affect juiciness, as meat from pigs reared indoors is found to be juicier than that from pigs reared outdoors [32]. In addition, center temperature significantly affects the juiciness of meat, as does the cooking procedure (heating time, temperature, and method); an increase in center temperature reduces the juiciness of meat [31].

#### 1.2.4. Flavor and Aroma

Meat flavor is an important attribute for consumers and highly correlated with overall consumer satisfaction [30]. Moreover, flavor is a complex sensory attribute that can be defined as a combination of taste components registered on the tongue and volatile compounds that flow through the nasal pathways to the odor epithelium in the nose [15].

Meat flavor is determined by different extrinsic and intrinsic factors of the animal from which the meat originated, including species, genetics, sex, feeding regimen, and management practices. Volatile compounds are the result of a combination of chemical processes that depend on both precursors in raw meat and the heating process [15].

The way meat is cooked can also influence flavor. Heating speed and cooking endpoint, cooking temperature, and the final fat level and composition can also influence flavor [33,34].

#### 1.2.5. Tenderness

Tenderness is one of the most critical attributes in terms of the overall acceptability of pork, although juiciness, flavor, and a lack of off-flavor are also significant for the general impression of meat quality [17].

To satisfy consumer preferences, the factors that determine tenderness should be optimized. Some of these factors are related to production methods, feeding, and genetics [22]. Nevertheless, other relevant factors occur postmortem and, if not optimized, in vivo factors are ineffective [35]. These factors include carcass refrigeration, aging time, cooking conditions, and temperature [36].

Based on the literature review above, we propose the following hypothesis:

**H1.**  *Sensory attributes (juiciness, flavor, aroma, and tenderness) directly impact the perceived overall quality of pork loin.*

### 1.3. Quality and Pig Breed

Pork meat is characterized by considerable variation in intrinsic attributes (color and fat content) depending on the breed, resulting in different quality experiences. This complicates the process of perceiving the quality of unprocessed pork and may induce consumers to search for other informative indicators in their quality assessment, such as the distinctive breed label.

The Duroc breed produces pork with a higher IMF content than white European breeds, including the Large White and Landrace [37]. Choi et al. [38] compared the carcass characteristics and meat quality of Duroc and crossbred pigs (Landrace × Yorkshire × Duroc; LYD). Their findings regarding subjective evaluation and sensory characteristics demonstrated that the Duroc breed had significantly higher scores than the crossbred pigs in all categories except tenderness. Channon et al. [39] analyzed the effect of the percentage of Duroc content on the meat and eating quality attributes of pork loin. Their findings showed that pork obtained from 100% Duroc pigs had 0.3–0.5% more IMF content. However, consumers did not report any benefits in terms of the quality attributes of the pork loin.

According to Choi et al. [38], all subjective evaluation items (marbling, texture, color, tenderness, juiciness, and flavor) were significantly higher in the Duroc population than in the crossbred pigs. According to the research results of Channon et al. [39], pork from 100% Duroc pigs was juicier and had higher intramuscular fat content than pork from 0 and 50% Duroc pigs.

Based on these findings, we propose the following hypothesis:

**H2.**  *Pig breed leads to differences in the perceived overall quality of pork loin.*

### 1.4. Quality and Product Familiarity

Familiarity is one of the most significant determinants of food product preferences because it reduces uncertainty about a product and results in a better match between expectations and product characteristics [40]. Familiarity with a product is thought to influence consumers’ interpretation of the information involved in their choice of product and assessment of product quality [7].

Banović et al. [7] segmented consumers according to their familiarity with specific beef cuts and found differences in their use of intrinsic and extrinsic cues in regard to their perception of beef quality. They concluded that consumers with a higher level of product familiarity considered the information they knew as the most relevant for evaluating a product, whereas consumers with a lower level of product familiarity were less capable of understanding cues that are relevant for inferring meat quality.

Borgogno et al. [8] examined the relationship of liking the appearance and taste of meat among consumers with different familiarity levels. They found that the high-familiarity group liked meat more regardless of the effects of storage or animals. Existing studies strongly support the concept that quality attributes may have different meanings depending on the degree of familiarity with a product.

The above discussion reveals that market segmentation is necessary to ensure that meaningful links can be created between products and consumers. Therefore, consumers’ consumption experiences and the positioning of different products within their preferences and consumption habits were considered.

Based on the above discussion, we formulated the following hypotheses:

**H3a.**  *Different consumer segments exist depending on familiarity with the pork loin product category.*

**H3b.**  *Familiarity with the pork loin product category directly impacts perceived overall quality.*

**H3c.**  *Familiarity with the pork loin product category moderates the relationship between pig breed and perceived overall quality.*

**Figure 1:**
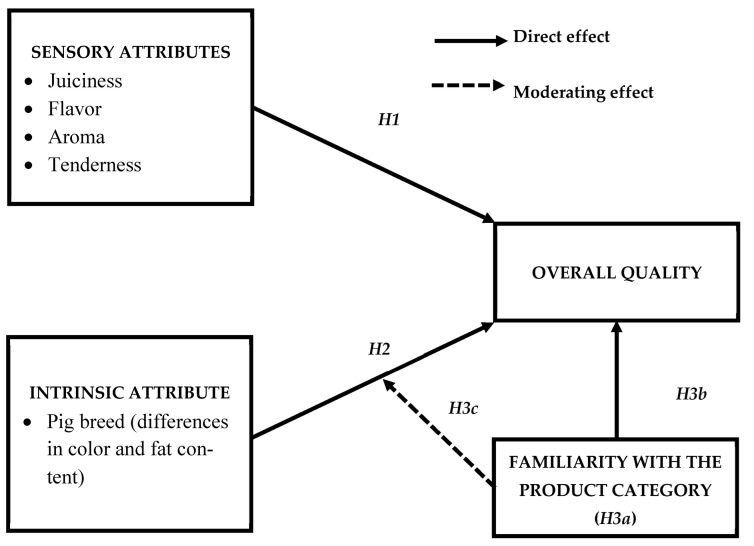
Proposed analytical model.

Figure 1 illustrates the proposed model, considering the theoretical antecedents described in the previous four subsections.

In sensory analysis, the null hypothesis (H0) typically states that there is no perceptible difference between samples or that any observed difference is due to random chance. Hence, the null hypothesis was formulated as follows:

**H0.**  *Differences in sensory and intrinsic attributes and familiarity with the product category do not influence consumer-perceived quality.*

## 2. Materials and Methods

### 2.1. Experimental Design and Data Collection

**Figure 2:**
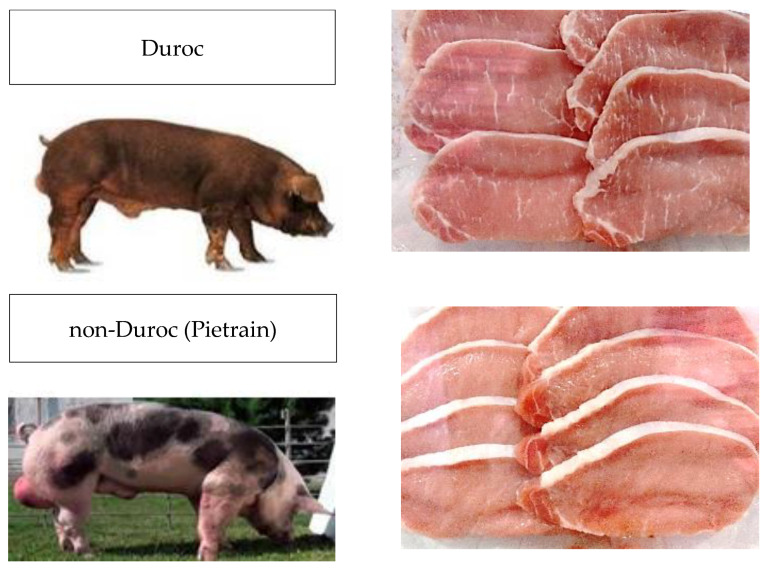
Differences in the pork loin samples based on the pig breed.

Data were collected in a tasting room in March 2024; the treatment of the samples was controlled by a group of researchers in food science and technology from the Faculty of Chemistry. In this study, we compared two pork loin samples (Duroc and non-Duroc). Specifically, the non-Duroc samples came from Pietrain pigs. Table 2 shows the physicochemical characteristics of the samples in relation to the color and percentage of IMF content. Figure 2 shows that the samples derived from pigs whose fathers were of the Duroc breed had redder tones and a higher IMF content.

All procedures performed in this study that involved human participants were in accordance with the ethical standards of the 1964 Helsinki Declaration and its later amendments or comparable ethical standards. The participants in our study were recruited using a convenience sampling method among adult consumers residing in a major city of Spain. Recruitment was carried out via personal contact and social networks, and participation was voluntary. The inclusion criteria required that the participants be over 18 years old, regular consumers of pork loin, and not have any dietary restrictions (vegetarian) or allergies that would interfere with the study. In the study, the quota controls were age, gender, and educational level.

All participants were required to provide an affirmative reply to participate in this survey, and informed consent was given via the statement “I am aware that my responses are confidential, and I agree to participate in this survey.” The study was explained to the participants in the tasting room. The participants were not financially compensated for their participation, and they were allowed to withdraw from the survey at any time without giving a reason. The products tested were safe for consumption.

In the first step, the participants entered the tasting room and were presented with two samples (Duroc and non-Duroc) of uncooked pork loin on trays. The samples were labeled and presented as they are found in the supermarket.

In the second step, the participants were randomly assigned to taste one of the two samples. In sensory analysis, a control group in the traditional sense of the word (as in other types of experiments) is not needed, but a reference or control sample is used to compare and evaluate the study samples. This “control” is not a separate untreated group, but a known sample that serves as a reference point to evaluate sensory differences. In our study, three controlled tasting groups were used. Each group conducted the tasting on a different day. The participants were randomly assigned to taste each of the two samples tested. The meat samples were cooked on a grill without salt or oil to avoid contaminating the original flavor. Tasters were provided with breadsticks and water. Then, the participants completed a structured questionnaire.

The questionnaire consisted of three sections. The first section contained sociodemographic information. The second section measured the sensory attributes perceived by the consumers, who rated each attribute on a 10-point semantic differential scale and the perceived overall quality on a 10-point semantic differential scale from (1) very low to (10) very high. The last section included items regarding familiarity with the product category, measured through shopping and cooking experiences (four items) and purchase and consumption frequencies (two items).

### 2.2. Sample Profile

A sample of 130 Spanish consumers were used in this study; 63.1% were women and 36.9% were men. The age distributions were 50.8% (18–34 years), 36.2% (35–54 years), and 13% (>54 years). Regarding purchase and consumption frequencies, the respondents indicated that they purchased (73.9%) and consumed pork loins (80%) at least once a fortnight.

Regarding the sample size, the number of participants (*n* = 130) is consistent with previous sensory and consumer studies in food science [41,42], where samples ranging from 100 to 150 consumers are commonly considered adequate for obtaining reliable hedonic and preference data.

### 2.3. Statistical Analysis

Hypotheses 1, 2, 3b and 3c were tested by estimating two analysis of covariance (ANCOVA) models, using overall perceived quality as the dependent variable. ANCOVA is a statistical method that combines elements of analysis of variance (ANOVA) and regression. ANCOVA is used when there is a dependent variable and a categorical independent variable. In this study, the dependent variable was perceived overall quality, and the categorical independent variables were juiciness, flavor, aroma, tenderness, and breed. The first ANCOVA model incorporated pig breed as a factor and the four sensory attributes as covariates, making it possible to test Hypotheses 1 and 2. The second ANCOVA model included two factors (pig breed and familiarity with the pork loin product category), their interaction (breed × familiarity), and the four covariates from the previous model. Therefore, the second model made it possible to test Hypotheses 3b and 3c by analyzing the estimated parameters associated with familiarity and breed × familiarity factors, respectively.

Additionally, latent class cluster analysis (LCCA) is a statistical segmentation technique that allows for the identification of hidden groups (latent classes) within a population from observed patterns of responses. It is particularly useful when the groups are not previously known and cannot be directly observed. In this study, LCCA was used to identify different clusters of consumers according to their familiarity with the pork loin product category (Hypothesis 3a). Therefore, in this case, six variables related to shopping and cooking experiences (four variables) and purchase and consumption frequencies (two variables) were used as indicators. Statistical analyses were performed using IBM SPSS Statistics 28.0 and Latent Gold^®^ 5.1.

## 3. Results

### 3.1. Impact of Sensory Attributes and Breed on Perceived Overall Quality

Table 3 shows that, regarding sensory attributes (Hypothesis 1), flavor, tenderness, and juiciness significantly impact perceived quality. The effect of aroma is not significant (*p* = 0.056). The effect of breed (Hypothesis 2), as an intrinsic attribute reflecting differences in color and fat content, is statistically significant (*p* < 0.05), and the perceived quality of the Duroc samples is lower than that of the non-Duroc samples (MDuroc = 6.94, MNon-Duroc = 7.42). Overall, this model explains approximately 65% of the variance in perceived overall quality.

### 3.2. Impact of Familiarity with the Product Category on Perceived Overall Quality

As previously mentioned, LCCA was used to identify different consumer segments according to their familiarity with the product category (Hypothesis 3a). The indicators used for segmentation purposes were shopping experience (SE1 and SE2), cooking experience (CE1 and CE2), and purchase and consumption frequencies. The results in Table 4 suggest the existence of three clusters based on consumer familiarity with this product category.

Table 5 shows that the six indicators helped segment consumers into three groups (*p* < 0.01). The percentage of variance explained ranges from 58.3% for purchase frequency to 16% for the second item related to the shopping experience.

The results in Table 6 enable the characterization of the three groups of identified consumers. Cluster 1 accounts for 41% of the sample and is denoted as “Frequent and expert consumers” because they had the highest scores in relation to their shopping and cooking experiences. Together with the consumers in Cluster 2, they were also the most frequent purchasers and consumers of pork loins. Cluster 2 represents 37% of the consumers surveyed and is referred to as “Frequent but inexperienced consumers” because, despite their high purchase and consumption frequencies, they attained the lowest scores for three of the four items related to the subjective perception of their shopping and cooking experiences. Finally, Cluster 3 accounts for 22% of the sample and is referred to as “Infrequent and inexperienced consumers” because they had the second lowest scores in shopping and cooking experience (ahead only of the consumers in Cluster 2), and the majority almost never purchased or consumed pork loin.

Finally, to test Hypotheses 3b and 3c, a second ANCOVA model was estimated by including a factor denominated as familiarity with three categories (as many as the previously identified consumer clusters), along with an interaction effect (breed × familiarity), to test the moderating effect. Table 7 shows that neither the familiarity factor nor the factor that included the interaction effect was statistically significant (*p* > 0.05). Therefore, familiarity does not affect perceived overall quality, nor does it moderate the importance of the breed during the assessment of overall quality.

## 4. Conclusions

Understanding the influence of the sensory and intrinsic attributes of meat on consumer acceptance and evaluation are imperative for ensuring consistent high-quality pork production that aligns with consumer preferences and elevates overall satisfaction levels. In this study, we provide a comprehensive overview of the diverse factors affecting pork meat quality, including sensory and intrinsic attributes.

The results indicate that Spanish consumers can detect differences in the quality of pork loin meat based on taste. More specifically, based on evaluations of sensory attributes by taste, the main determinant of perceived quality is flavor, followed by tenderness and juiciness. These results are consistent with those of Miller [18].

Considering the results as a whole, there is sufficient evidence to conclude that sensory and intrinsic attributes determine significant differences in the quality perceived by consumers. Essentially, the evidence suggests that the observed results are unlikely to have been produced solely by random and, consequently, the null hypothesis is rejected.

Therefore, Hypothesis 1, which states that sensory attributes directly impact perceived overall quality, is partially supported, as aroma does not significantly influence perceived quality. One possible explanation for this effect is proposed by Van Ba et al. [43]. Their findings highlight that meat flavor and aroma develop principally during cooking because of the complex interaction of precursors derived from both the lean and fat compositions of meat, which produce volatile components. Thousands of volatile components have been detected and identified, some of which are extensively described in previous studies, such as those by Van Ba et al. [43] and Soriano et al. [44].

Our results concur with those of Żakowska-Biemans et al. [45]. Their findings highlight that Polish consumers prefer low-fat meat and are unwilling to buy meat containing IMF because they associate it with a higher caloric value and cholesterol content.

The results related to the breeds tested (Duroc versus non-Duroc) indicate that the perceived quality of Duroc pork is valued to a lesser extent than that of other breeds, despite having greater marbling and a more intense color. The results support Hypothesis 2 and are consistent with those of Ngapo et al. [20] and Font-I-Furnols et al. [26], who concluded that most consumers prefer light-red pork and minimal fat coverage.

In general, greater marbling can negatively affect consumers’ evaluation of quality because it is perceived as unhealthy. It has been reported that the amount of visible IMF or marbling negatively affects consumers purchase decision, most likely due a perception of it being less healthy than lean meat [46,47,48]. Brewer et al. [49] found that chops with less than 2.5% IMF had a higher overall appearance acceptability and purchase intent than chops with higher IMF content (3.0% to 3.5%). Fernandez et al. [50] also found that purchasing intention decreased with increasing IMF level. Our results are in line with those from Garmatyk et al. [51], who found that the Pietrain breed has less intramuscular fat content, and hence a lower calorific value for its meat.

Consumers are more aware of the link between high fat intake and various health issues, such as heart disease and obesity. This has led to a greater demand for leaner protein sources, including pork. While some consumers enjoy the flavor and texture of fatty cuts, many prefer the leaner options for both health reasons and perceived weight management benefits. In essence, the trend toward leaner pork is a reflection of consumers’ growing awareness of the relationship between diet and health, combined with changes in pork production that have made leaner options more readily available. The Spanish consumer, similar to consumers elsewhere, is concerned about fatty pork as a potentially unhealthy product. Shan et al. [52] reported similar findings in a study focused on Irish consumers; more recently, similar results were obtained by Chernukha et al. [53] in a study on Russian consumers. Verbeke et al. [21] formulated another possible explanation for this result by stating that good knowledge and training are required to appreciate marbling. Therefore, based on the segmentation performed in this study, only 41% of the evaluated consumers can be considered experienced.

Regarding color, our results are in line with those of Zhang et al. [54], who compared five breeds of pigs and concluded that there is a relationship between breed and meat color. Specifically, differences are caused by variations in hemoglobin content and myoglobin forms. Moreover, a higher content of intramuscular fat results in different values for CIELAB scale parameters.

For Hypothesis 3, three clusters of consumers were identified according to their degree of familiarity with the pork loin product category. These three clusters differed regarding their shopping and cooking experiences, as well as their purchase and consumption frequencies. Therefore, Hypothesis 3a is supported. To the best of our knowledge, this study is the first to identify consumer segments based on their familiarity with the pork loin product category in the context of the Spanish market. However, despite confirming the existence of heterogeneity in the Spanish market according to consumer familiarity, no empirical support was found for Hypotheses 3b and 3c. Furthermore, differences in familiarity do not directly influence the assessment of overall perceived quality nor moderate the relationship between pig breed and overall perceived quality. Therefore, the importance of pig breed is equal regardless of consumers’ level of familiarity, which is an important implication for producers and distributors. According to our findings, it is crucial to promote the importance of aspects such as breed and feeding and rearing systems through information and promotion campaigns, so that consumers can become familiar with the relationship between the attributes described above and the final quality of the product.

The results of this study show that consumers prefer meat with low IMF content and light-colored meat. A better understanding of this perception may help improve the competitiveness of the pork industry through effective product innovation and labeling strategies. Competitive strategies in the meat industry should be directed toward a combination of innovations in healthier products and labels that provide more detailed information on the nutritional and health properties of the products.

At the marketing level, health properties play an increasingly important role as determining factors in food purchase decisions. Therefore, innovations in the nutritional and health properties of pork products must be carefully tested through market research to identify the most suitable products.

Regarding limitations, it should be stated that this study was conducted with a sample of Spanish consumers, which may limit the generalizability of the findings to broader populations with different cultural backgrounds, dietary habits, or socio-economic conditions. While a consumer segmentation approach was employed to account for heterogeneity within the sample, it may not fully capture the diversity of the Spanish population, particularly in terms of age distribution, income levels, or regional consumption patterns. Furthermore, the sensory evaluation in this study focused on sensory and intrinsic attributes, and other potentially influential attributes, such as visual appearance, granularity of texture, and aftertaste, were not assessed. These factors may also contribute to the overall perception of meat quality and consumer satisfaction. Notably, while aroma was included in the evaluation, it did not show a significant impact on perceived quality. This outcome may be partially explained by methodological limitations related to aroma detection under the specific testing conditions, which might not have allowed for full aroma development or accurate sensory perception by participants.

Based on the findings of this study, future research should aim to explore several avenues to deepen the understanding of consumer preferences in pork meat consumption. First, it would be valuable to extend this research to a broader and more diverse international sample to assess the cross-cultural generalizability of the observed patterns, particularly regarding sensory preferences and health-related perceptions of intramuscular fat. Additionally, incorporating a wider range of sensory attributes, such as visual appeal, texture complexity, and aftertaste, could provide a more comprehensive understanding of the multidimensional nature of perceived meat quality. Given the limited influence of aroma observed in this study, further investigations under alternative sensory testing conditions that better replicate real cooking environments may yield more accurate assessments of aroma’s role in consumer evaluations. Moreover, future studies should adopt longitudinal or experimental designs to evaluate how consumer preferences evolve over time, especially in response to educational interventions or marketing strategies that promote knowledge of product origin, breed characteristics, and nutritional content. Finally, considering psychological and behavioral factors, such as food neophobia, health consciousness, and trust in labeling, may offer a more holistic framework to guide both academic research and industry practice in aligning pork production with dynamic consumer expectations.

## Figures and Tables

**Table 1:** Main types of quality attributes in the consumer behavior literature.

TYPE OF QUALITY ATTRIBUTES	
**SEARCH ATTRIBUTES**	
**Definition**	Attributes typically used at the point of sale to evaluate alternative choices. They are known as “quality cues”. Divided into [6,9,10]: Intrinsic cues: These are inherent visible characteristics of the product. These attributes allow for measuring quality objectively and give the product its functionality. Extrinsic cues: These include information related to the product but that is not physically part of the product. They are strongly associated with it and must be considered in any evaluation of the product’s characteristics.
**Cost of quality detection** **(pre/post-purchase)**	Low pre-cost of quality detection. Allow the buyer to compare and identify the best quality alternative by a simple examination.
**Specific attributes**	Cut type, color, fat structure/type (marbling/rim fat), freshness, and visible fat. Quality labels (including brands and quality assured symbols), place of purchase, packaging, price, promotion, designation of origin, information about animal feed, production, and processing.
**EXPERIENCE ATTRIBUTES**	
**Definition**	Elements of product quality that can be experienced during consumption [9].
**Cost of quality detection** **(pre/post-purchase)**	High pre-cost but low post-cost of quality detection. The buyer obtains the quality information as a by-product of post-purchase consumption. This information contributes to decision making on repeat purchases.
**Specific attributes**	Taste, tenderness, juiciness, flavor, and convenience.
**CREDENCE ATTRIBUTES**	
**Definition**	Attributes that consumers cannot assess either before or after the purchase but need to be communicated [11].
**Cost of quality detection** **(pre/post-purchase)**	High pre-cost and high post-cost of quality detection. The buyer must be reliant on third-party expertise or on the seller’s credentials.
**Specific attributes**	Animal welfare, product safety, organic feed, health claims (hormones, antibiotics), environmentally friendliness, and egalitarian claims.

**Table 2:** Physicochemical characteristics of the color and IMF content of the pork loin samples.

Samples	Red Color (CIELAB Coordinate)	IMF Content (Grams per 100 g)
**Duroc**	3.18 ^b^	3.12 ^b^
**Non-duroc**	2.42 ^a^	1.86 ^a^

(a,b) Different superscript letters (a,b) in the same column indicate significant differences between the mean values, as obtained by Student *t*-test (*p* < 0.05).

**Table 3:** Impact of sensory attributes and pig breed on perceived overall quality.

Attribute	*F*	*p*	Partial η^2^	β
**Intercept**	11.951	<0.001	0.088	1.587
**Juiciness**	5.550	0.020	0.043	0.156
**Flavor**	27.496	<0.001	0.181	0.356
**Aroma**	3.721	0.056	0.029	0.108
**Tenderness**	10.479	0.002	0.078	0.207
**Breed**	4.456	0.037	0.035	−0.324 ^a^

Notes: Levene’s test of equality of error variances (*F* = 0.038, *p* = 0.845); corrected model (*F* = 45.150, *p* < 0.001, R2 = 0.645, adjusted R2 = 0.631); ^a^ Parameter for “Duroc”.

**Table 4:** Fit indices for the latent class cluster models tested: relative fit indices and classification-based information criterion.

Model	Log-Likelihood (LL)	BIC (LL)	CAIC (LL)	ICL-BIC	Number of Parameters	Classification Errors
**1 cluster**	−1507.905	3234.502	3279.502	3234.502	45	0.000
**2 clusters**	−1454.150	3161.010	3213.010	3204.637	52	0.071
3 clusters *	−1424.622	3135.973	3194.973	3190.699	59	0.082
**4 clusters**	−1410.274	3141.296	3207.296	3205.569	66	0.096
**5 clusters**	−1400.423	3155.612	3228.612	3232.994	73	0.118

Notes: BIC: Bayesian information criterion; CAIC: consistent Akaike information criterion; ICL-BIC: integrated classification likelihood criterion estimated using a BIC-type approximation; * best model according to BIC, CAIC, and ICL-BIC.

**Table 5:** Estimated parameters for the solutions obtained for the three clusters: indicators.

Indicator	Cluster 1	Cluster 2	Cluster 3	Robust Wald Statistic	*p*	*R* ^2^
**SE1.** I am sufficiently familiar with the product category of pork loin	0.644	−0.385	−0.259	17.191	<0.001	0.405
**SE2.** I am well acquainted with the different brands of pork loin offered in the shops	0.270	−0.038	−0.232	12.012	0.003	0.160
**CE1.** When I cook pork loin at home, I am the one who does the cooking	0.464	−0.325	−0.139	18.144	<0.001	0.354
**CE2.** I prepare pork loins using different recipes	0.525	−0.315	−0.210	6.147	0.046	0.369
**Purchase frequency**	−0.804	−0.943	1.747	6.833	0.033	0.583
**Consumption frequency**	−0.777	−0.798	1.576	8.181	0.017	0.534

**Table 6:** Profiles for the three clusters of consumers.

Indicator	Cluster 1. Frequent and Expert Consumers (41%)	Cluster 2. Frequent but Inexperienced Consumers (37%)	Cluster 3. Infrequent and Inexperienced Consumers (22%)
**SE1**	8.03	4.92	5.40
**SE2**	5.26	3.79	3.03
**CE1**	8.76	5.23	6.42
**CE2**	7.99	4.68	5.21
**Purchase frequency**			
Several times per week	**12%**	**14%**	0%
Once a week	**49%**	**51%**	1%
Once every 15 days	**31%**	**28%**	8%
Once a month	7%	6%	**23%**
Almost never	2%	1%	**65%**
Never	0%	0%	**3%**
**Consumption frequency**			
Almost every day	**4%**	**4%**	0%
Several times per week	**20%**	**20%**	0%
Once a week	**46%**	**46%**	3%
Once every 15 days	**27%**	**26%**	19%
Once a month	3%	2%	**19%**
Almost never	1%	1%	**60%**

Notes: For shopping and cooking experiences, the values marked with different colors indicate statistically significant differences in means among the clusters based on paired comparison analyses. The highest percentages per row for the purchase and consumption frequencies are shown in bold.

**Table 7:** Impact of sensory attributes, pig breed, and familiarity (direct and moderating effect) on perceived overall quality.

Attribute	*F*	*p*	Partial η^2^
**Intercept**	9.995	0.002	0.077
**Juiciness**	5.122	0.025	0.041
**Flavor**	28.090	<0.001	0.191
**Aroma**	3.391	0.068	0.027
**Tenderness**	10.243	0.002	0.079
**Breed**	3.988	0.048	0.028
**Familiarity**	1.445	0.240	0.024
**Breed × familiarity**	0.258	0.773	0.004

Notes: Levene’s test of equality of error variances (*F* = 1.343, *p* = 0.251); corrected model (*F* = 25.267, *p* < 0.001, R2 = 0.656, adjusted R2 = 0.630).

## Data Availability

The original contributions presented in the study are included in the article, further inquiries can be directed to the corresponding author.

## Abbreviations

The following abbreviations are used in this manuscript:

ANCOVA	Analysis of covariance
BIC	Bayesian information criterion
CAIC	Consistent Akaike information criterion
CIELAB	Commission Internationale de l’Éclairage as the organization defining this color space. L (color luminosity), a (green-red color coordinate), b (blue-yellow color coordinate).
ICL-BIC	Integrated classification likelihood criterion estimated using a BIC-type approximation
IMF	Intramuscular fat
LCCA	Latent class cluster analysis
LL	Log-likelihood
Ph	Potential of hydrogen
